# Sleep restriction during pregnancy and its effects on blood pressure and renal function among female offspring

**DOI:** 10.14814/phy2.12888

**Published:** 2016-08-22

**Authors:** Rogério Argeri, Erika E. Nishi, Rildo A. Volpini, Beatriz D. Palma, Sergio Tufik, Guiomar N. Gomes

**Affiliations:** ^1^ Department of PhysiologyEscola Paulista de Medicina – UNIFESPSão PauloBrazil; ^2^ Department of PsychobiologyEscola Paulista de Medicina – UNIFESPSão PauloBrazil; ^3^ Basic Research Laboratory – LIM12Nephrology – Faculty of MedicineUSP, São PauloSão PauloBrazil; ^4^ Centro Universitário São CamiloSão PauloBrazil

**Keywords:** Blood pressure, female offspring, renal function, sleep restriction

## Abstract

The influence of sleep restriction (SR) during pregnancy on blood pressure and renal function among female adult offspring was investigated. Pregnant Wistar rats were distributed into control and SR groups. The SR was performed between the 14th and 20th days of pregnancy (multiple platforms method for 20 h/day). At 2 months of age, half of the offspring from both groups were subjected to an ovariectomy (ovx), and the other half underwent sham surgery. The groups were as follows: control sham (C_sham_), control ovx (C_ovx_), SR sham (SR_sham_), and SR ovx (SR_ovx_). Renal function markers and systolic blood pressure (BPi, indirect method) were evaluated at 4, 6, and 8 months. Subsequently, the rats were euthanized, kidneys were removed, and processed for morphological analyses of glomerular area (GA), number of glomeruli per mm^3^ (NG), and kidney mass (KM). Increased BPi was observed in the C_ovx_, SR_sham_, and SR_ovx_ groups compared to C_sham_ at all ages. Increased plasma creatinine concentration and decreased creatinine clearance were observed in the SR_sham_ and SR_ovx_ groups compared to the C_sham_ and C_ovx_ groups. The SR_ovx_ group showed higher BPi and reduced creatinine clearance compared to all other groups. The SR_ovx_ group showed reduced values of GA and KM, as well as increased NG, macrophage infiltration, collagen deposit, and ACE1 expression at the renal cortex. Therefore, SR during pregnancy might be an additional risk factor for developing renal dysfunction and increasing BP in female adult offspring. The absence of female hormones exacerbates the changes caused by SR.

## Introduction

During critical stages of intrauterine growth, environmental alterations may predispose the fetus to the development of diseases later in life (Frasch et al. 2007). This concept has been confirmed by several studies in which nutritional deficiency (Regina et al. 2001; Franco et al. 2009), diabetes (Rocha et al. 2005; de Almeida Chaves Rodrigues et al. 2013), and the use of cortisol (Ortiz et al. 2003; Celsi et al. 1998) during pregnancy are related to the development of cardiovascular and metabolic diseases in the offspring at adulthood (Barker 1997; Langley‐Evans et al. 1999; Alexander et al. 2015).

The reduction of the sleep period has become commonplace in modern society (Spiegel et al. 1999; Aldabal and Bahammam 2011). However, sleep restriction (SR) is associated with augmented production of hormones, such as corticosterone, growth hormone (GH), ACTH, and inflammatory cytokines (Spiegel et al. 1999; Schüssler et al. 2006; Aldabal and Bahammam 2011). Consequently, the reduction of sleep time during pregnancy may alter the fetal environment and may also be a threat to maternal and fetal health (Chang et al. 2010; Pires et al. 2010; Pardo et al. 2016).

During pregnancy, physical and hormonal changes affect sleep quality (Sahota et al. 2003; Lopes et al. 2004; Pien and Schwab 2004). Additionally, if these changes occur alongside long working hours (Caruso 2006; National Sleep Foundation, 2007), the probability of a pregnant woman having sleep disorders increases.

In the past decade, SR has been the subject of several studies; however, the impact of this condition during pregnancy on the offspring has been evaluated in only a few studies. The effect of SR during the critical period for kidney growth (the last week of pregnancy for rats) was studied by Thomal et al. (2010). In this study, the authors observed that male offspring from SR mothers presented increased blood pressure and reduced nephron number (Thomal et al. 2010). Additionally, Lima et al. showed altered sensitivity of the cardiac baroreflex response and increased blood pressure (BP) in male offspring of rats subjected to SR throughout the entire pregnancy (Lima et al. 2014).

Taking into account that few studies have focused on the repercussions of SR during pregnancy on female offspring, this study aims to evaluate the effects of SR over the last week of pregnancy on blood pressure, renal morphology, and function among female offspring at adulthood. Additionally, we evaluated the expression of ACE1 and ACE2 in the kidneys in view that altered expression of RAS components have been related to renal modifications in fetal programming models (Sahajpal and Ashton 2005; Mesquita et al. 2010; Gwathmey et al. 2011). Considering that the effects of female hormones on cardiovascular and renal function could cover up SR effect over these systems, we also evaluated the effects of SR in females submitted to ovariectomy.

## Methods

This experimental protocol was approved by the Ethical Research Committee of the Universidade Federal de São Paulo – UNIFESP (CEUA: 7647020614) and adhered to international guidelines for the care of research animals.

Female Wistar rats weighing between 200 and 250 g and male Wistar rats weighing between 300 and 350 g were used in this study. The animals had free access to food and water during the entire experimental protocol and were maintained in a room with fixed temperature (22°C) and cycle of light (light and dark cycle alternating every 12 h, starting the lighting period at 7 am). Two females were caged overnight with a male, and vaginal smears were collected the following morning. The presence of sperm was regarded as a positive result and was considered as day 0 of pregnancy. The females were then distributed in two experimental groups: control and sleep‐restricted mothers. Seventeen mothers composed the control group. Twenty‐six mothers composed the SR group.

### Sleep restriction protocol

The sleep restriction technique was based on the muscle atony that accompanies paradoxical sleep (Jouvet et al. 1964; Thomal et al. 2010). Briefly, 10 narrow circular platforms (6.5 cm in diameter) were placed inside a tiled tank (123 × 44 × 44 cm) filled with water to within 1 cm below the upper border of the platform. For the sleep‐restricted mothers group, 2–6 rats were placed on the platforms in an arrangement that allowed them to move inside the tank and jump from one platform to the other. Two days before the beginning of the study, the animals were adapted to the water tank for a period of 1 h to avoid unnecessary drops in the water. The sleep‐restricted mothers group was placed in the tank between the 14th and 20th days of pregnancy for 20 h/24 h (from 2 pm h until next day at 10 am). Following sleep restriction period, rats were placed back in their home cages and could sleep freely. At 10 am on the 20th day of pregnancy, the sleep‐restricted mothers were placed back in their home cages for maintenance until spontaneous parturition and weaning of the offspring. The animals of the control group remained in their home cages in the same room in which the sleep restriction occurred. The sleep restriction was carried out between the 14th and 20th days of pregnancy because, in rats, the last week of pregnancy is critical for the kidney development (Nigam et al. 1996), and so is more susceptible to changes in maternal environment.

### Birth and weaning

After birth, the animals were weighed, and the litters were reduced to six offspring, preferably in the ratio of four males to two females; these pups were left with the mother for 28 days. Following the weaning period, the offspring were separated from their mothers; female offspring were placed in collective cages containing four animals per cage. The males were used in another study. The offspring were divided into two groups: control offspring (C) and offspring from sleep‐restricted mothers (SR).

### Experimental groups

At 2 months, one female of each litter (C and SR) was submitted to ovariectomy (OVX) and the other underwent to sham surgery. The rats were anesthetized with a combination of xylazine (10 mg/kg) and ketamine (75 mg/kg) given intraperitoneally (i.p.). The surgery was performed using the transverse incision technique as described by Saadat Parhizkar and Latiff (2008). The groups were as follows: control sham (C_sham_), control ovx (C_ovx_), SR sham (SR_sham_), and SR ovx (SR_ovx_).

### Body weight

The offspring were weighed after birth and at 4, 6, and 8 months of age on an analytical balance.

### Evaluation of ovariectomy and adiposity

Considering that in the absence of female hormones, the uterine body undergoes atrophy (Naciff et al. 2007), after euthanizing the animals, the uterus was removed and weighed to confirm ovariectomy. In addition, the retroperitoneal fat pads were bilaterally collected and weighted as an index of adiposity.

### Indirect determination of systolic blood pressure (BPi)

At 3 months of age, the offspring began the adaptation to the tail plethysmography apparatus (IITC Life Science, Woodland Hills, CA). Effective determinations of indirect systolic blood pressure (BPi) were obtained at 4, 6, and 8 months of age. The animals were placed in acrylic cylinders with appropriate dimensions for the size of the animal while the tail remained exposed. The sphygmomanometer with a sensor connected to a register system was then adjusted to the proximal tail portion of the rat. Three measurements were performed in sequence; the mean of these three measurements was considered the BPi.

### Renal function

Renal function evaluations were performed at 4, 6, and 8 months of age. The rats were placed in metabolic cages for 24 h. Urine and blood samples were collected to measure creatinine, sodium, and potassium concentrations. Plasma and urine creatinine levels were measured using the Jaffé method, and the glomerular filtration rate (GFR) was determined based on creatinine clearance. The concentrations of sodium and potassium were measured using flame photometry (Analyser 910, SP/SP, Brazil). The quantification of urinary protein concentration was performed using the Bradford method and urine osmolarity was measured using an osmometer Advanced 3W2 (Advanced Instruments Inc., Norwood, MA).

### Renal morphology

Kidney samples from rats of all groups were fixed in Bouin's solution (ethanol saturated with picric acid 75%, formaldehyde 20%, and acetic acid 5%) and were processed for paraffin embedding. Five‐micron histological sections were stained with hematoxylin–eosin for morphology or with picrosirius red for interstitial collagen type I and III analyses.

Glomerular area and number were evaluated using a light microscope (Nikon H550L) with a camera connected to a computer with image analysis software (Nikon, NIS‐Elements 3.2, Japan). Encircled areas were determined by computerized morphometry. Analyses of morphological procedures were performed by an investigator who was blinded to the origin of the slides. Twenty‐five fields were analyzed on each slide (magnification 200×). For the interstitial collagen type I and III analyses, five fields were analyzed on each slide (magnification 100×). Images were acquired with a digital camera (AxioCam MRc) connected to a Carl Zeiss microscope with polarized light. Collagens I and III were estimated as percentages using the image analysis software ImageJ (NIH, USA).

### Immunohistochemistry and immunofluorescence

ED1‐positive cells (macrophages/monocytes) were identified by immunohistochemistry. Tissue sections were incubated for 12 h at 4°C with a monoclonal anti‐ED1 antibody (1:1000, Serotec, Oxford, UK). The reaction product was determined with a streptavidin peroxidase complex (Dako LSAB System HRP, DAKO Corporation, Carpinteria, CA). The material was counterstained with Carazzi's hematoxylin, dehydrated, and mounted. Macrophage infiltration was evaluated using a Nikon microscope at a magnification of 200×. Each studied field had an area of 275,000 μm^2^.

Angiotensin‐converting enzymes type 1 and 2 (ACE1 and ACE2) expressions were evaluated by immunofluorescence. Tissue sections were incubated for 12 h at 4°C with a mouse anti‐rat ACE1 monoclonal antibody (1:500 ab11738, Abcam, Cambridge, UK) or with rabbit anti‐rat ACE2 monoclonal antibody (1:100 ab108252, Abcam, Cambridge, UK), followed by incubation at room temperature for 1 h with an Alexa Fluor^®^ 488‐conjugated anti‐mouse secondary antibody (1:500 dilution, Molecular Probes, Thermo Fisher Scientific, Waltham, MA) or an Alexa Fluor^®^ 594‐conjugated anti‐rabbit secondary antibody (1:500 dilution, Molecular Probes, Invitrogen). Sections were then coverslipped with mounting medium containing DAPI (Sigma‐Aldrich, St. Louis, MO). Fluorescent images (magnification 630×) of the renal cortex (5 fields/rat) were collected using a Leica SP8 confocal microscope system. For these images, the laser power was set at 20%, and the excitation/emission wavelengths were 488 nm/band pass 530–555 nm for Alexa Fluor 488 and 405 nm/band pass 440–480 nm for DAPI. ACE1 and ACE2 fluorescence intensities were measured using ImageJ software (NIH, USA).

The results are presented as the means ± standard error (SEM). Statistical tests used were Student's *t*‐test and one‐way and two‐way analysis of variance (ANOVA) when appropriate (GraphPad Prism software 5^®^). The level of significance was 5% (*P* ≤ 0.05). After one‐way ANOVA, the Bonferroni test was used for multiple comparison.

## Results

### Effects of SR on body weight

**Figure 1:**
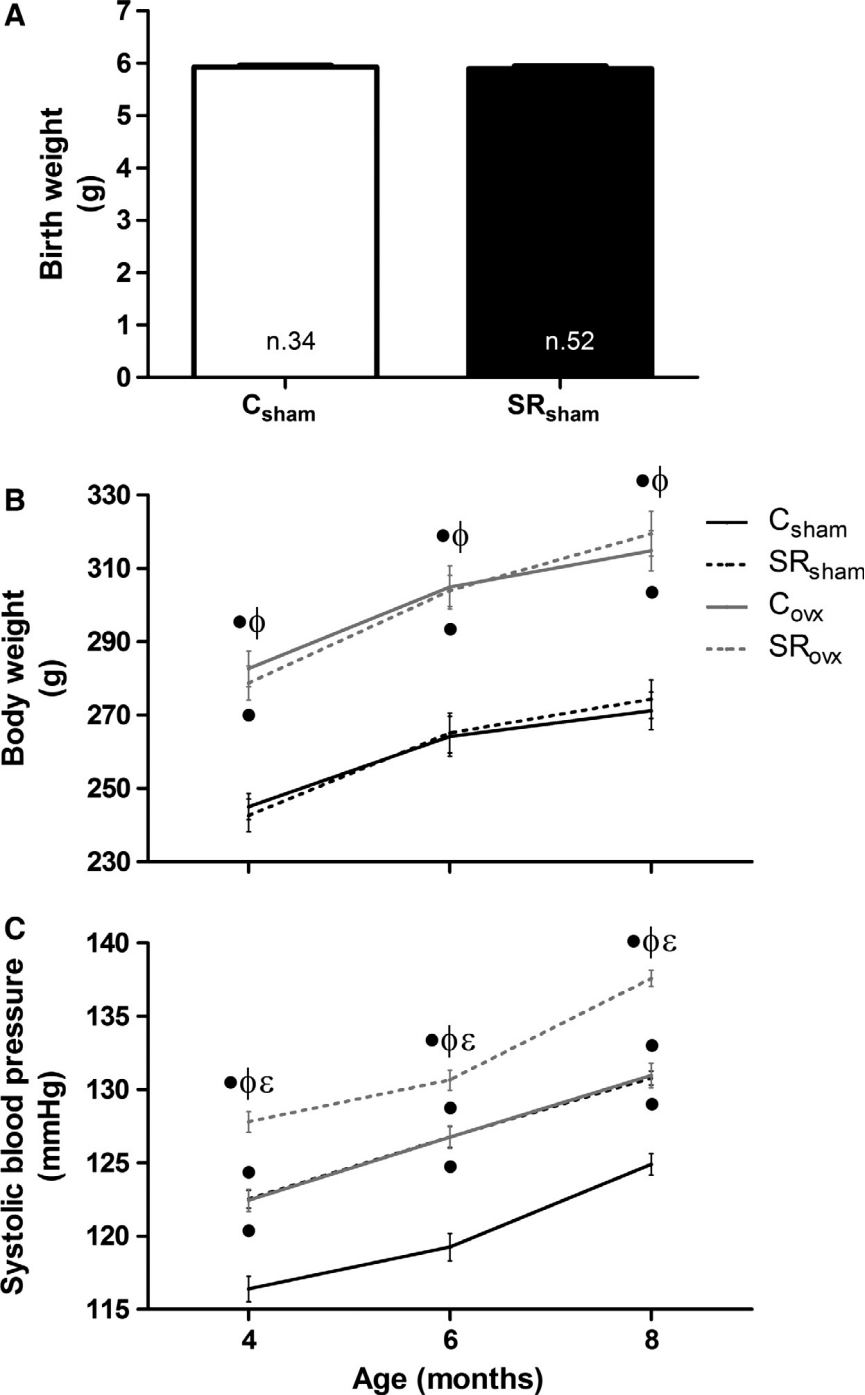
Body weight (A and B) and systolic blood pressure (C) in the offspring, submitted to ovariectomy surgery (_OVX_) or not (_Sham_), of control (C) and sleep‐restricted (SR) mothers. ^●^ *P* < 0.05 versus C_S__ham_; ^ɸ^ *P* < 0.05 versus SR_S__ham_; ^Ɛ^ *P* < 0.05 versus C_ovx_ (Bonferroni test).

There was no significant difference in birth weight between the groups (C: 5.92 ± 0.034 [*n* = 34]; SR: 5.90 ± 0.04 [*n* = 52]). However, from 4 months on, the ovariectomized animals showed increased body weight compared to controls (Fig. 1).

At 8 months, the amounts of retroperitoneal fat were augmented in C_ovx_ and SR_ovx_ rats compared to C_sham_ and SR_sham_ rats, suggestive of greater adiposity in ovariectomized rats (C_sham_: 3.7 ± 0.3 g [*n* = 15]; C_ovx_: 6.8 ± 0.6 g [*n* = 15]; SR_sham_: 3.8 ± 0.4 g [*n* = 13]; SR_ovx_: 8.7 ± 0.9 g [*n* = 15]) (two‐way ANOVA: OVX > Sham, *P* = 0.0001).

### Ovariectomy assessment

The uterine weight was measured to confirm ovariectomy efficacy. Ovariectomized animals had reduced uterine weights compared to the sham groups (C_sham_: 1.3 ± 0.1 g [*n* = 17]; C_ovx_: 0.21 ± 0.01 g [*n* = 17]; SR_sham_: 1.0 ± 0.05 g [*n* = 26]; SR_ovx_: 0.19 ± 0.01 g [*n* = 26]). The SR_sham_ group had decreased uterine weight compared to C_sham_ (two‐way ANOVA: OVX < Sham, *P* = 0.0001; SR < C, *P* = 0.0024).

### Effects of SR on blood pressure and renal function

Higher values of BPi were observed in the C_ovx_, SR_sham_, and SR_ovx_ groups compared to the C_sham_ group (Fig. 1C). The SR_ovx_ group had higher BPi compared to all of the other experimental groups at all ages. At 8 months, the BP of the C_sham_ group was 124.9 ± 0.74 mmHg. At the same age, BP of C_ovx_ and SR_sham_ groups were about 6 mmHg greater and in SR_ovx_ group was about 12.7 mmHg greater than the C_sham_.

The values for creatinine plasma concentration and creatinine clearance are shown in Table 1. Increased creatinine plasma concentration and decreased creatinine clearance were observed in the SR_sham_ and SR_ovx_ groups compared to the C_sham_ and C_ovx_ groups at all ages studied. Yet, at 8 months, the SR_ovx_ group had decreased clearance of creatinine compared to the SR_sham_ group.

**Table 1:** Creatinine plasma concentration and creatinine clearance (Clcr) in the offspring, submitted to ovariectomy surgery (_OVX_) or not (_Sham_), of control (C) and sleep‐restricted (SR) mothers

Groups	Creatinine (mg/dL)	Clcr (mL/min/kg)	Two‐way ANOVA
4 months			
C_sham_ (*n* = 17)	0.53 ± 0.02	4.64 ± 0.21	Clcr C > SR
SR_sham_ (*n* = 26)	0.63 ± 0.02*	3.46 ± 0.17*	*P* = 0.001
C_ovx_ (*n* = 17)	0.54 ± 0.02	4.39 ± 0.15	Creatinine C < SR
SR_ovx_ (*n* = 26)	0.65 ± 0.02*, ‡	3.21 ± 0.14*, ‡	*P* = 0.001
6 months			
C_sham_ (*n* = 17)	0.50 ± 0.02	4.81 ± 0.23	Clcr C > SR
SR_sham_ (*n* = 26)	0.58 ± 0.02*	3.81 ± 0.19*	*P* < 0.001
C_ovx_ (*n* = 17)	0.50 ± 0.03	4.75 ± 0.43	Creatinine C<SR
SR_ovx_ (*n* = 26)	0.59 ± 0.02*, ‡	3.58 ± 0.20*, ‡	*P* = 0.001
8 months			
C_sham_ (*n* = 17)	0.53 ± 0.01	4.56 ± 0.30	Clcr C > SR (*P* = 0.001)
SR_sham_ (*n* = 26)	0.64 ± 0.02*	3.37 ± 0.18*	OVX ≠ Sham (*P* = 0.005)
C_ovx_ (*n* = 17)	0.55 ± 0.02	4.09 ± 0.32	Creatinine C < SR
SR_ovx_ (*n* = 26)	0.68 ± 0.03*, ‡	2.48 ± 0.17*, †, ‡	*P* = 0.001

Significance level: *P < 0.05 versus C_sham_; ^†^ *P* < 0.05 versus SR_sham_; ^‡^ *P* < 0.05 versus C_ovx_ (Bonferroni test). Values are means ± standard error. *n*, number of animals.

The plasma sodium and potassium concentrations were in normal ranges in all studied groups, without significant changes. The excreted loads (EL) of sodium and potassium were similar in the studied groups at 4 and 6 months, shown in Table 2. However, at 8 months, there was a significant reduction in the EL of sodium and potassium in the SR_ovx_ group compared to the C_sham_ and C_ovx_ groups.

**Table 2:** Plasma (P) sodium and potassium concentrations and excreted loads (EL) of sodium and potassium in the offspring, submitted to ovariectomy surgery (_OVX_) or not (_Sham_), of control (C) and sleep‐restricted (SR) mothers

Groups	P_Na_^+^ mEq/L	EL_Na_^+^ mEq/L/24 h	P_K_^+^ mEq/L	EL_K_^+^ mEq/L/24 h	Two‐way ANOVA
4 months					
C_sham_ (*n* = 17)	140.8 ± 0.5	1.19 ± 0.06	3.24 ± 0.03	1.84 ± 0.08	
SR_sham_ (*n* = 26)	143.5 ± 0.7	1.09 ± 0.05	3.37 ± 0.05	1.65 ± 0.08	
C_ovx_ (*n* = 17)	141.3 ± 0.7	1.18 ± 0.07	3.32 ± 0.09	1.88 ± 0.08	
SR_ovx_ (*n* = 26)	142.8 ± 0.7	1.05 ± 0.06	3.38 ± 0.05	1.55 ± 0.1	
6 months					
C_sham_ (*n* = 17)	142.5 ± 0.5	1.26 ± 0.1	3.32 ± 0.07	1.86 ± 0.1	
SR_sham_ (*n* = 26)	144.5 ± 0.9	1.21 ± 0.05	3.37 ± 0.07	1.64 ± 0.06	
C_ovx_ (*n* = 17)	143.3 ± 0.9	1.33 ± 0.05	3.36 ± 0.07	1.90 ± 0.07	
SR_ovx_ (*n* = 26)	144.9 ± 0.8	1.15 ± 0.06	3.46 ± 0.09	1.59 ± 0.1	
8 months					
C_sham_ (*n* = 17)	143.8 ± 0.9	1.14 ± 0.05	3.31 ± 0.08	1.80 ± 0.07	EL_Na_^+^ SR < C
SR_sham_ (*n* = 26)	145.8 ± 1.1	1.06 ± 0.05	3.52 ± 0.06	1.54 ± 0.06	(*P* = 0.008)
C_ovx_ (*n* = 17)	146.0 ± 0.8	1.13 ± 0.06	3.46 ± 0.09	1.84 ± 0.08	EL_K_^+^ SR < C
SR_ovx_ (*n* = 26)	147.4 ± 0.7	0.89 ± 0.07*, †	3.58 ± 0.08	1.34 ± 0.09*, †	(*P* = 0.0001)

Significance level: *P < 0.05 versus C_sham_; ^†^ *P* < 0.05 versus C_ovx_ (Bonferroni test).

Values are expressed as mean ± standard error. *n*, number of animals.

The values of urinary flow, urinary osmolarity, and proteinuria are shown in Table 3. At 4 and 8 months of age, there was a reduction in urinary flow in the SR_ovx_ group compared to the C_sham_ and SR_sham_ groups. No significant changes were observed in the other parameters.

**Table 3:** Urinary flow, urinary osmolarity, and proteinuria for 24 h in 4, 6, and 8 months old offspring, submitted to ovariectomy surgery (_OVX_) or not (_Sham_), of control (C) and sleep‐restricted (SR) mothers

Groups	Urinary flow (mL/min/kg)	Urinary Osm (mOsm/KgH_2_O)	Proteinuria (mg/24 h)	Two‐way ANOVA
4 months				
C_sham_ (*n* = 17)	0.029 ± 0.001	2445.1 ± 80.5	4.42 ± 0.25	Urinary flow
SR_sham_ (*n* = 26)	0.028 ± 0.002	2257.7 ± 86.9	5.55 ± 0.32	SR < C (*P* = 0.048)
C_ovx_ (*n* = 17)	0.028 ± 0.002	2416.8 ± 70.2	4.61 ± 0.31	OXV < Sham (*P* = 0.015)
SR_ovx_ (*n* = 26)	0.022 ± 0.001*, †	2152.5 ± 95.2	5.08 ± 0.36	
6 months				
C_sham_ (*n* = 17)	0.025 ± 0.001	2232.4 ± 87.4	4.92 ± 0.38	
SR_sham_ (*n* = 26)	0.027 ± 0.001	2020.8 ± 78.4	5.49 ± 0.35	
C_ovx_ (*n* = 17)	0.023 ± 0.002	2193.2 ± 86.6	4.72 ± 0.35	
SR_ovx_ (*n* = 26)	0.023 ± 0.002	1968.0 ± 62.2	5.46 ± 0.43	
8 months				
C_sham_ (*n* = 17)	0.025 ± 0.002	2112.0 ± 75.5	5.02 ± 0.41	Urinary flow
SR_sham_ (*n* = 26)	0.027 ± 0.002	1902.8 ± 75.6	6.49 ± 0.52	OXV < Sham (*P* = 0.0008)
C_ovx_ (*n* = 17)	0.023 ± 0.003	2040.0 ± 76.6	4.75 ± 0.32	
SR_ovx_ (*n* = 26)	0.018 ± 0.001*, †	1869.9 ± 83.6	5.64 ± 0.55	

Significance level: *P < 0.05 versus C_sham_; ^†^ *P* < 0.05 versus SR_sham_ (Bonferroni test).

Values are expressed as mean ± standard error. *n*, number of animals.

### Effect of SR on kidney morphology

The morphological parameters are presented in Table 4. The glomerular area values obtained for the SR_ovx_ were significantly smaller than those observed in the C_sham_ and SR_sham_ groups. Yet, an increased number of glomeruli per mm^3^ was observed in the SR_ovx_ group. The kidney cross‐sectional area was reduced in the C_ovx_ compared to the C_sham_ group and in the SR_ovx_ group in relation to the C_sham_ and SR_sham_ groups. Kidney weight was also significantly decreased in the C_ovx_ and SR_sham_ groups compared to the C_sham_ group and in the SR_ovx_ group in relation to the C_sham_, C_ovx_, and SR_sham_ groups.

**Table 4:** Glomerular area, number of glomeruli, kidney cross‐sectional area, kidney cortical area, and kidney weight in the offspring, submitted to ovariectomy surgery (_OVX_) or not (_Sham_), of control (C) and sleep‐restricted (SR) mothers

Groups	C_sham_	SR_sham_	C_ovx_	SR_ovx_	Two‐way ANOVA
Glomerular area (μm^2^)	7936 ± 106 (388)	7831 ± 108 (404)	7654 ± 104 (444)	7365 ± 87*, † (492)	OVX < Sham (*P* = 0.0002) SR < C (*P* = 0.05)
Number of glomeruli/mm^3^	91 ± 2 (6)	96 ± 5 (6)	106 ± 11 (6)	119 ± 6* (6)	OVX > Sham (*P* = 0.008)
Kidney cross‐sectional area (mm^2^)	104.6 ± 1.7 (6)	100.4 ± 1.5 (6)	97.0 ± 2.0* (6)	90.3 ± 1.8*, ‡ (6)	SR < C (*P* = 0.006) OVX < Sham *P* = 0.0001)
Kidney cortical area (%)	59.2 ± 0.5 (6)	58.6 ± 0.4 (6)	58.5 ± 0.3 (6)	58.2 ± 0.5 (6)	
Kidney weight/100 g bw	0.72 ± 0.02 (17)	0.65 ± 0.01* (26)	0.58 ± 0.01* (17)	0.53 ± 0.01*, †, ‡ (26)	SR < C (*P* = 0.0001) OVX < Sham *P* = 0.0001)

Significance level: *P < 0.05 versus C_sham_; ^†^ *P* < 0.05 versus SR_sham_; ^‡^ *P* < 0.05 versus C_ovx_ (Bonferroni test).

Values are means ± standard error. *n*, number of animals. In the glomerular area, the number in parentheses refers to the quantity of glomeruli analyzed.

**Figure 2:**
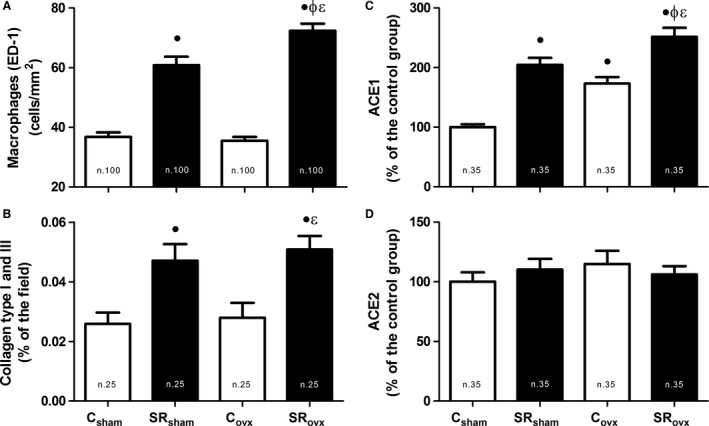
Macrophages (ED‐1) expression (A), collagen type I and III expression (B), ACE1 expression (C), and ACE2 expression (D) in renal tissue of the offspring, submitted to ovariectomy surgery (_OVX_) or not (_Sham_), of control (C) and sleep‐restricted (SR) mothers. ^●^ *P* < 0.05 versus C_S__ham_; ^ɸ^ *P* < 0.05 versus SR_S__ham_; ^Ɛ^ *P* < 0.05 versus C_ovx_ (Bonferroni test). The number of fields analyzed is shown inside the bars.

**Figure 3:**
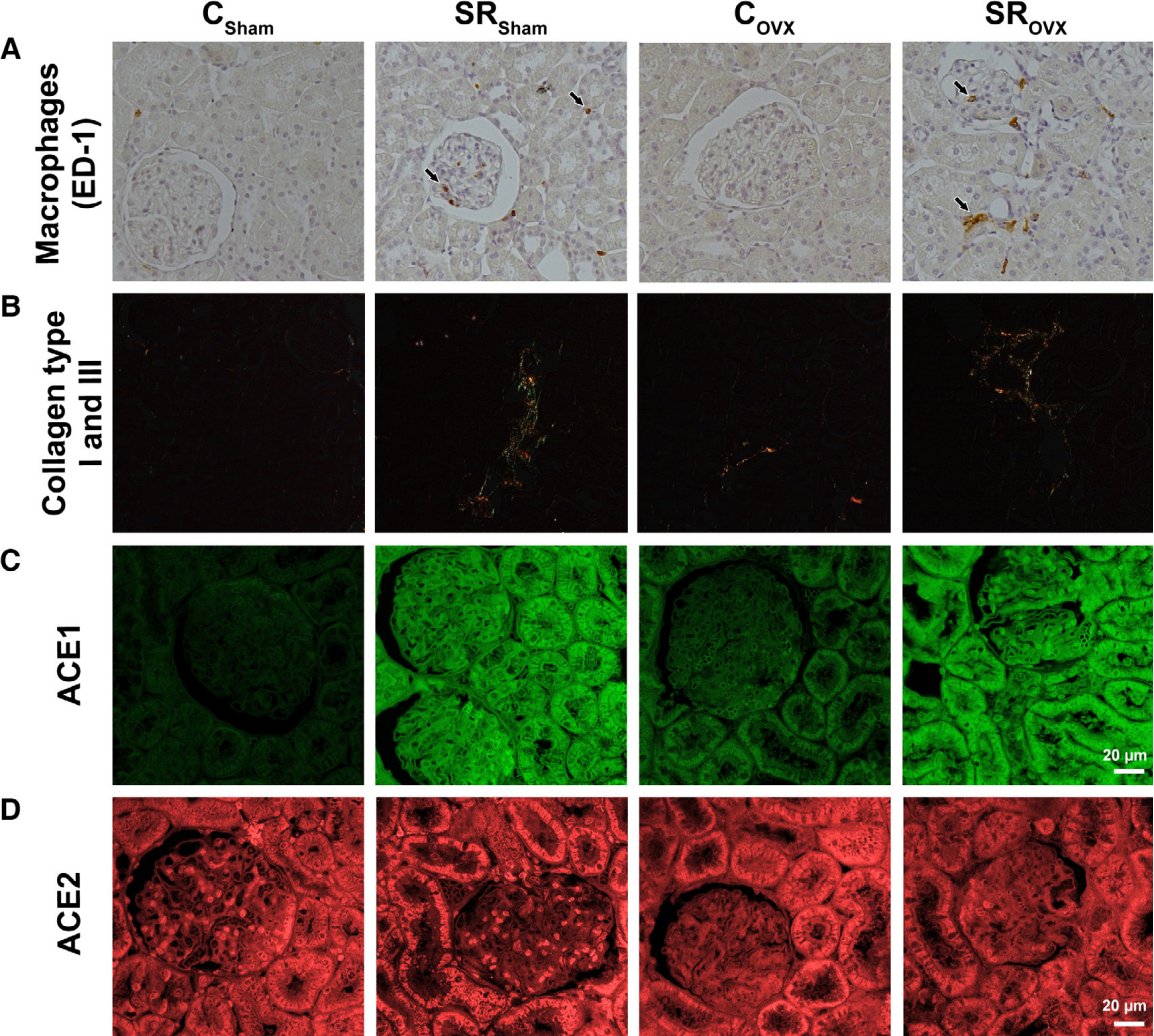
Representative photomicrographs of the renal tissue in the offspring, submitted to ovariectomy surgery (_OVX_) or not (_Sham_), of control (C) and sleep‐restricted (SR) mothers. (A) Macrophages (ED‐1) marked in brown color (original magnification: 200×). (B) Collagen type I and III (Picrosirius red – polarized light – original magnification: 100×). (C) ACE1 and (D) ACE2 expression by immunofluorescence (original magnification: 630×).

Figure 2 shows the expression of factors related to renal impairment. The presence of macrophages in renal tissue (Fig 2A and 3A) was increased in the SR_sham_ and SR_ovx_ groups compared with the C_sham_ and C_ovx_ groups. The presence of collagen types I and III in the renal interstitium (Figs 2B and 3B) increased significantly in the SR_sham_ and SR_ovx_ groups compared to the C_sham_ and C_ovx_ groups. ACE1 expression (Figs 2C and 3C) was increased in the C_ovx_, SR_sham_, and SR_ovx_ groups compared to the C_Sham_ group. OVX in the SR group produced even higher expression of ACE1 expression. There was no significant difference in ACE2 expression among the groups.

## Discussion

In this study, the consequences of SR in late pregnancy on the morphological and functional aspects of the kidneys in female offspring were evaluated. Renal changes and higher blood pressure were observed in female offspring from SR mothers. The SR groups, with or without oophorectomy, showed significant reduction in creatinine clearance at the studied ages, suggesting that prenatal changes resulting from SR predispose to renal dysfunction. Ovariectomy enhanced the elevation of blood pressure caused by SR during pregnancy and at 8 months of age, renal changes were more pronounced in SR_ovx_ group (decreased: creatinine clearance, urinary flow, and excreted sodium load).

The removal of ovaries resulted in increased body weight and fat deposition in both offspring of C and SR. In addition, in SR, the ovariectomy caused significant morphological renal changes: (1) reduction in glomerular area, (2) increase in the number of glomeruli per mm^3^, probably related to (3) significant reductions in cross‐sectional area of kidney and renal mass.

In female rat, there are interactions between the estrogen (E), thyroid hormones (TH), and growth hormone (GH) (Giustina and Veldhuis 1998), and the absence of E leads to obesity by reducing the action these hormones (Ignacio et al. 2012). Our results of body weight and fat deposition in OVX groups are in agreement with previous studies (Giustina and Veldhuis 1998; Ignacio et al. 2012; Pijacka et al. 2015). Additionally, we observed reduction of renal mass in C_ovx_, SR_sham_, and SR_ovx_ groups (more pronounced in SR_ovx_ group). The reduction of renal mass after ovariectomy was also observed by Pijacka et al. (2015) in rats subjected to protein restriction during fetal development. In these animals, ovariectomy was associated with greater renal damage shown by significant reduction in creatinine clearance and renal mass, changes suggestive of accelerated renal aging. Thus, it is possible that sleep restriction during pregnancy also modifies renal morphology and function of female offspring and after ovariectomy, the alterations were more evident.

Hypertension and cardiovascular disease resulting from an adverse environment during fetal development have been associated with reduced renal mass and nephron number (Kett and Denton 2011); however, these changes are not common in all studies (Bertram et al. 2011; Ortiz et al. 2003; Singh et al. 2007). In 1988, Brenner et al. postulated that reduced filtering surface area due to fewer nephrons leads to sodium retention and hypertension (Brenner et al. 1988; Luyckx et al. 2013). However, other mechanisms may also be involved in the development of hypertension in adults (Oparil et al. 2003). In this study, the SR did not reduce the nephron number in female offspring, though it did cause a reduction in renal mass, suggesting changes in kidney development. Ovariectomy accentuated the reduction in renal growth, which may have led to the relative increase in the number of glomeruli per mm^3^. However, even with more glomeruli, a loss of kidney function and an increase in blood pressure were apparent.

Sleep restriction during pregnancy resulted in increased blood pressure in both genders; however, the male rat offspring had reduced nephron numbers (Thomal et al. 2010) which did not occur in female offspring, at the same experimental protocol. Studies in rats show that stress during pregnancy increases maternal cortisol production altering fetal development (Mairesse et al. 2007; Amugongo and Hlusko 2014). Increased maternal corticosteroids affects the offspring in a sex‐specific manner especially with regard to placental levels of the enzyme 11 beta‐hydroxysteroid dehydrogenase type 2 (11*β*‐HSD‐2), enzyme that protect the fetus from maternal glucocorticoid excess (Cuffe et al. 2012). Thus, it is possible that changes in 11*β*‐HSD‐2 expression levels in the placentas of males and females are related to the different results obtained at morphological analysis. However, further experiments are needed to confirm this hypothesis.

The higher BP values observed in groups subjected to ovariectomy were expected as female hormones, especially estrogen, are considered cardiovascular protective factors in premenopausal women (Murphy 2011). The main vascular effects of estrogen are vasodilatation, reduced vascular inflammation, and improved vascular reactivity (Murphy 2011). These effects occur through the activation of estrogen receptors (ERs), which can be nuclear, non‐nuclear, and G‐protein coupled (GPER or GPR30) (Murphy 2011; Prossnitz and Barton 2011). The vasodilatory effect of estrogen is mediated mainly through the generation of nitric oxide (NO), as this effect is abolished after the use of NO synthesis inhibitors (Bucci et al. 2002).

Concerning renal function, the beneficial effects of female sex hormones also seem to be associated with NO production (Dubey and Jackson 2001; Nielsen et al. 2003). Physiologically, NO interacts with the renin–angiotensin system (RAS), and estrogen changes the ratio between NO and ANG II, leading to a larger proportion of intrarenal NO/ANG II (Nielsen et al. 2003). Renal changes in the expression of RAS components have been described in the model of prenatal exposure to betamethasone, namely, augmented AT1 receptor expression and decreased AT2 receptor expression in the renal cortices of young sheep offspring (Gwathmey et al. 2011). Similar results were observed in the offspring of rats subjected to protein restriction (Sahajpal and Ashton 2005; Mesquita et al. 2010). These studies suggest that insults during pregnancy lead to changes in renal RAS, which likely contribute to cardiovascular changes in the offspring.

In our model, increased ACE1 expression occurred in parallel with the increase in blood pressure and the greatest increase in ACE1 expression and BP were observed when both treatments were combined (SR and ovariectomy – ovx). Differently from the results of ACE1, the expression of ACE2 was not changed in the experimental groups. These results suggest that SR during pregnancy results in renal imbalance of RAS resulting in increased blood pressure. In the absence of female hormones the interaction between NO and RAS is weakened, aggravating the changes caused by SR. The kidneys from SR groups (SR_sham_ and SR_ovx_), in addition to the increase in ACE1 expression, showed increased macrophage infiltration and collagen type I and III expression. These results suggest that in these groups, aside from RAS alterations, other factors appear to contribute to the decline of renal function. Hypertension itself is an important factor in the induction of renal disease (Ruiz‐Ortega et al. 2006); in this way, it is difficult to take apart the influence of high blood pressure from the effects of sleep restriction during pregnancy on the renal changes observed in this experimental model.

Sleep restriction and deprivation alter the production and secretion of several hormones (Spiegel et al. 1999; Schüssler et al. 2006; Pardo et al. 2016). According to Plagemann et al. (1999) during critical ontogenetic periods, hormones such as insulin, leptin, and others, once present in nonphysiological concentrations, can act as “endogenous functional teratogens” responsible for the onset of hypothalamic dysfunction in the offspring (Plagemann et al. 1999; Tzschentke and Plagemann 2006). In this theory, hormones play a central role, as they are subject to influences from the environment and also modulate the neuroendocrine and immune systems, which, in turn, regulate all key processes in the body (Dörner 1974, 1989). Thus, it is possible that sleep restriction during pregnancy results in hormonal changes that affects renal development and additionally may also modify the hypothalamic–pituitary–adrenal axis and, thereby, alters the hormonal balance of offspring in adulthood.

## Conclusion

Sleep restriction imposed at the end of pregnancy affects female offspring, causing changes that result in high blood pressure and kidney abnormalities in adulthood. Ovariectomy exacerbates the changes caused by sleep restriction. Additional studies are needed to understand the mechanisms responsible for the changes observed in this study.

## Conflict of Interest

None declared.
